# Supplementary material for: Defining the order of assembly of the Clostridioides difficile divisome complex
Source: J Bacteriol. 2026 Aug 27;208(9):e00032-26. doi: 10.1128/jb.00032-26 (PMC13584725; doi:10.1128/jb.00032-26)
Supplement: Supplementary file 1 [file jb.00032-26-s0001.docx]

**SUPPLEMENTARY FIGURES AND TABLES**

**Fig S1: Growth and morphology of the Δ*ylmG*, Δ*ylmH*, and Δ*divIVA* mutants.** (A) The indicated strains were cultured to mid-log phase and then inoculated into 96-well plates and their OD_600_ was monitored over time. The mean and standard error of n = 4 biological replicates are shown. (B-C) The cell shape of the indicated strains was quantified using SuperSegger to determine the cell length (B) and cell width (C) across the cell population. Light blue dots indicate a single cell whereas larger dark blue symbols represent the mean of an individual replicate. In B-C >2,400 cells were quantified across 2-4 different replicates. An ordinary one-way ANOVA with Tukey’s post-test was used to test for statistically significant differences. Only statistically significant comparisons are shown, all other comparisons were not significant. **** p<0.0001; *** p<0.001; ** p<0.01; ns, not significant.

**Fig S2: Quantification of the mid-cell enrichment of divisome proteins.** (A) To quantify the fold-enrichment of divisome proteins at the mid-cell relative to the sidewall/cytoplasm, ≥200 cells were identified and the maximum fluorescence at the site of septum synthesis was quantified relative to the maximum fluorescence of a section of the sidewall and/or cytoplasm using FIJI. The colors represent independent experiments, where each dot indicates a cell and the larger symbols represent the median mid-cell enrichment value for each experiment; the mean and standard deviation between replicates are shown. Statistically significant differences were determined using a lognormal ordinary one-way ANOVA with Tukey’s post-test. **** p<0.0001; ns, not significant. For cytosolic proteins (FtsZ, ZapA, SepF, MldB, MldC, and DivIVA), values greater than one indicate mid-cell enrichment over the sidewall/cytoplasm. However, for transmembrane proteins (PBP1, MldA, FtsK, and PBP3), enrichment would be theoretically indicated by a value greater than two, since there are two membranes at the site of division once septum synthesis has completed. To account for this, we generated a mScI3-fusion to the first transmembrane helix of the *E. coli* MalF protein to serve as a non-specific transmembrane control (mScI3-TM). As the enrichment values for mScI3-PBP1, mScI3-FtsK, and mScI3-PBP3 are not statistically significantly increased compared to mScI3-TM, they are not specifically enriched at the mid-cell relative to the sidewall, suggesting that these proteins are evenly distributed throughout the cell and/or that they may also have a role in sidewall synthesis. (B) *C. difficile* expressing constructs encoding the indicated protein-mScI3 fusions under the control of an aTc-inducible P*_tet_* promoter from an ectopic site were treated with 5 ng/mL aTc for 1 hr. Note that this concentration of aTc is higher than that used for most constructs in Fig 2-7 to enable better detection of the fusion protein and degradation products. Cell lysates were prepared from an equivalent amount of bacteria, and western blotting using an anti-mScarlet antibody was performed, with anti-glutamate dehydrogenase (GDH) as a loading control. The * indicates a non-specific band, and cleaved products representing liberated mScI3 are indicated. All proteins include a (GGGGS)_3_ linker, except mScI3-TM, mScI3-MldA, mScI3-MldB, and mScI3-MldC fusion proteins which include a GSAGSAAGSGKL linker.

**Fig S3: Conditional expression of *mScI3*-*zapA* causes an unusual septal morphology.** Cells were treated as in Fig 3. When endogenous *zapA* expression is knocked down and *mScI3*-*zapA*_im_ is induced, the majority of septa have a normal appearance (Fig 3C); however, occasional “spirals” of HADA labeling were also observed. The spirals could be consistent with incomplete bundling of the FtsZ filaments by mScI3-ZapA during septum synthesis.

**Fig S4: Growth of CRISPRi-KD mutants.** (A) *C. difficile* harboring the pIA33-based CRISPRi-KD cassette with either a non-targeting sgRNA (neg) or the indicated KD construct were cultured in mid-log phase in the presence of 2.5% xylose for 3 hr to pre-deplete the pool of FtsZ, ZapA, SepF, or PBP1 before back-diluting into a 96-well plate and monitoring the OD_600_ over time. The mean and standard error between replicates are indicated, n = 4. The *ftsZ*-KD and *sepF*-KD achieve a higher OD_600_ than the negative control, likely due to the filaments that form settling into an opaque pellet at the bottom of the wells. (B-D) To test if *mScI3-pbp1* encodes a functional protein, we compared the growth of strains harboring the plasmid-encoded *pbp1*-KD construct alone (B) to strains with both the *pbp1*-KD construct and chromosomally encoded P*_tet_*-*pbp1*_im_ (C) or P*_tet_-mScI3-pbp1*_im_ (D) cultured in the presence of aTc and 2.5% xylose to induce the complementation constructs. All strains were pre-cultured in 2.5% xylose for 3 hr to pre-deplete the endogenous PBP1. Note that the same data for the non-targeting control (neg) in panels B-D is represented in each panel for comparison purposes. (C) Over-expression of *pbp1*_im_ induces toxicity, consistent with our prior results (1, 2). (D) Over-expression of *mScI3-pbp1*_im_ causes even more toxicity compared to the untagged variant, perhaps due to altered turnover of the tagged protein. Regardless, at 1 ng/mL aTc, the *mScI3*-*pbp1*_im_ construct almost fully complements the growth inhibition caused by *pbp1*-KD, demonstrating that the fusion protein is functional within a narrow expression range.

**Fig S5: ZapA contributes to the proper assembly of SepF foci.** (A) The number of rings/foci was quantified per μm of cell length for 50 cells per replicate across 2 replicates. The small dots indicate individual cells or filaments, while the larger symbols represent the mean across all cells from each replicate. Independent replicates are color-coded, and the mean and standard deviation between replicates are indicated. A lognormal ordinary one-way ANOVA with Tukey’s post-test was used to determine statistical significance: **** p<0.0001; *** p<0.001; ns, not significant. A lognormal test was chosen to account for the >1-log difference in means across samples. (B) The width of rings/foci was quantified for >100 foci per replicate across two replicates. The small dots indicate individual cells or filaments, while the larger symbols represent the median from each replicate. Independent replicates are color-coded, and the mean and standard deviation between replicates are indicated. The *zapA*-KD prevents efficient FtsZ-mScI3 ring formation, so foci width was not quantified in this strain (nd). An ordinary one-way ANOVA with Tukey’s post-test was used to determine statistical significance: *** p<0.001; ** p<0.01; ns, not significant. (C) Representative images of key strains are shown. White represents mScI3 signal, and blue represents the HADA labeling, scale bars = 5 μm. The HADA signal is normalized across all images, but the mScI3 signal is scaled independently for SepF-mScI3, FtsZ-mScI3, and mScI3-ZapA to enhance visibility of the foci for each fusion. (D) A model to explain the observed localization in panels A-C and in **Fig 4**. We find that *ftsZ*-KD prevents localization of PBP1, SepF, and ZapA. In contrast, *zapA*-KD prevents localization of PBP1 and FtsZ, but SepF forms distinct foci, although the foci are decondensed and irregular. The *sepF*-KD strain still forms normal FtsZ and ZapA foci, but lacks PBP1, and the *pbp1*-KD strain still forms normal FtsZ/ZapA/SepF foci.

**Fig S6: Growth and cell width of non-essential divisome protein deletion mutants.** (A-B) The indicated strains were cultured to mid-log and then inoculated into a 96-well plate; the OD_600_ was monitored over time. The mean and standard error between replicates are shown, n = 4 biological replicates. Note that the WT data are the same between panels and reproduced in both panels for comparison purposes. (C) SuperSegger was used to quantify cell width across >2,500 total cells from n = 2-4 biological replicates. The light green dots indicate individual cells, whereas the larger dark green dots represent the average cell length from a single replicate. The mean and standard error of the mean between replicates are indicated. An ordinary one-way ANOVA with Tukey’s post-test was used to determine statistical significance. All comparisons were not statistically significant (ns).

**Table S1: *C. difficile* strains used in this study**

| Strain # | Strain Name (Description) | Source |
| --- | --- | --- |
| 756 | 630Δ*erm* Δ*pyrE* (Erythromycin-sensitive derivative of 630 with a deletion in *pyrE*) | (3) |
| 4825 | 630Δ*erm* *pyrE^+^* (#756 with *pyrE* restored) | (3) |
| 3747 | 630Δ*erm* pIA33-*sepF* | this work |
| 4960 | 630Δ*erm* pIA33-*divIVA* | this work |
| 3741 | 630Δ*erm* pIA33-*ftsZ* | this work |
| 5428 | 630Δ*erm* pIA33-*zapA* | this work |
| 4918 | 630Δ*erm* pIA33-*pbp1* | this work |
| 4926 | 630Δ*erm* pIA33-neg | this work |
| 4659 | 630Δ*erm pyrE*^+^*::*P*_tet_-ftsZ-(GGGGS)_3_-mScI3* | (1) |
| 5272 | 630Δ*erm pyrE*^+^*::*P*_tet_-mScI3-(GGGGS)_3_-zapA* | this work |
| 4553 | 630Δ*erm pyrE*^+^*::*P*_tet_-sepF-(GGGGS)_3_-mScI3* | this work |
| 4759 | 630Δ*erm pyrE*^+^*::*P*_tet_-mScI3-(GGGGS)_3_-pbp1* | (1) |
| 4672 | 630Δ*erm pyrE*^+^*::*P*_tet_-ftsZ*_im_ + pIA33-*ftsZ* | this work |
| 4676 | 630Δ*erm pyrE*^+^*::*P*_tet_-ftsZ*_im_*-(GGGGS)_3_-mScI3* + pIA33-*ftsZ* | this work |
| 5509 | 630Δ*erm pyrE*^+^*::*P*_tet_-zapA*_im_ + pIA33-*zapA* | this work |
| 5507 | 630Δ*erm pyrE*^+^*::*P*_tet_-mScI3-(GGGGS)_3_-zapA*_im_ + pIA33-*zapA* | this work |
| 4680 | 630Δ*erm pyrE*^+^*::*P*_tet_-sepF*_im_ + pIA33-*sepF* | this work |
| 4684 | 630Δ*erm pyrE*^+^*::*P*_tet_-sepF*_im_*-(GGGGS)_3_-mScI3* + pIA33-*sepF* | this work |
| 4737 | 630Δ*erm pyrE*^+^*::*P*_tet_-pbp1*_im_ + pIA33-*pbp1* | this work |
| 4739 | 630Δ*erm pyrE*^+^*::*P*_tet_-mScI3-(GGGGS)_3_-pbp1*_im_ + pIA33-*pbp1* | this work |
| 5002 | 630Δ*erm pyrE*^+^*::*P*_tet_-ftsZ-(GGGGS)_3_-mScI3 +* pIA33-neg | this work |
| 5430 | 630Δ*erm pyrE*^+^*::*P*_tet_-ftsZ-(GGGGS)_3_-mScI3 +* pIA33-*zapA* | this work |
| 4686 | 630Δ*erm pyrE*^+^*::*P*_tet_-ftsZ-(GGGGS)_3_-mScI3* + pIA33-*sepF* | this work |
| 4688 | 630Δ*erm pyrE*^+^*::*P*_tet_-ftsZ-(GGGGS)_3_-mScI3 +* pIA33-*pbp1* | this work |
| 5546 | 630Δ*erm pyrE*^+^*::*P*_tet_-mScI3-(GGGGS)_3_-zapA +* pIA33-neg | this work |
| 5548 | 630Δ*erm pyrE*^+^*::*P*_tet_-mScI3-(GGGGS)_3_-zapA +* pIA33-*ftsZ* | this work |
| 5933 | 630Δ*erm pyrE*^+^*::*P*_tet_-mScI3-(GGGGS)_3_-zapA +* pIA33-*sepF* | this work |
| 5552 | 630Δ*erm pyrE*^+^*::*P*_tet_-mScI3-(GGGGS)_3_-zapA +* pIA33-*pbp1* | this work |
| 5006 | 630Δ*erm pyrE*^+^*::*P*_tet_-sepF-(GGGGS)_3_-mScI3 +* pIA33-neg | this work |
| 4960 | 630Δ*erm pyrE*^+^*::*P*_tet_-sepF-(GGGGS)_3_-mScI3 +* pIA33-*ftsZ* | this work |
| 5432 | 630Δ*erm pyrE*^+^*::*P*_tet_-sepF-(GGGGS)_3_-mScI3 +* pIA33-*zapA* | this work |
| 4692 | 630Δ*erm pyrE*^+^*::*P*_tet_-sepF-(GGGGS)_3_-mScI3 +* pIA33-*pbp1* | this work |
| 4998 | 630Δ*erm pyrE*^+^*::*P*_tet_-mScI3-(GGGGS)_3_-pbp1 +* pIA33-neg | this work |
| 4772 | 630Δ*erm pyrE*^+^*::*P*_tet_-mScI3-(GGGGS)_3_-pbp1 +* pIA33-*ftsZ* | this work |
| 5434 | 630Δ*erm pyrE*^+^*::*P*_tet_-mScI3-(GGGGS)_3_-pbp1 +* pIA33-*zapA* | this work |
| 4774 | 630Δ*erm pyrE*^+^*::*P*_tet_-mScI3-(GGGGS)_3_-pbp1 +* pIA33-*sepF* | this work |
| 5525 | 630Δ*erm pyrE*^+^*::*P*_tet_-mScI3-GSAGSAAGSGKL-mldA* | this work |
| 5569 | 630Δ*erm pyrE*^+^*::*P*_tet_-mScI3-GSAGSAAGSGKL-mldA +* pIA33-*ftsZ* | this work |
| 5571 | 630Δ*erm pyrE*^+^*::*P*_tet_-mScI3-GSAGSAAGSGKL-mldA +* pIA33-*sepF* | this work |
| 5572 | 630Δ*erm pyrE*^+^*::*P*_tet_-mScI3-GSAGSAAGSGKL-mldA +* pIA33-*pbp1* | this work |
| 5538 | 630Δ*erm pyrE*^+^*::*P*_tet_-mScI3-GSAGSAAGSGKL-mldB* | this work |
| 5574 | 630Δ*erm pyrE*^+^*::*P*_tet_-mScI3-GSAGSAAGSGKL-mldB +* pIA33-*ftsZ* | this work |
| 5576 | 630Δ*erm pyrE*^+^*::*P*_tet_-mScI3-GSAGSAAGSGKL-mldB +* pIA33-*sepF* | this work |
| 5577 | 630Δ*erm pyrE*^+^*::*P*_tet_-mScI3-GSAGSAAGSGKL-mldB +* pIA33-*pbp1* | this work |
| 5527 | 630Δ*erm pyrE*^+^*::*P*_tet_-mScI3-GSAGSAAGSGKL-mldC* | this work |
| 5579 | 630Δ*erm pyrE*^+^*::*P*_tet_-mScI3-GSAGSAAGSGKL-mldC +* pIA33-*ftsZ* | this work |
| 5581 | 630Δ*erm pyrE*^+^*::*P*_tet_-mScI3-GSAGSAAGSGKL-mldC +* pIA33-*sepF* | this work |
| 5582 | 630Δ*erm pyrE*^+^*::*P*_tet_-mScI3-GSAGSAAGSGKL-mldC +* pIA33-*pbp1* | this work |
| 5033 | 630Δ*erm pyrE*^+^*::*P*_tet_-divIVA-(GGGGS)_3_-mScI3* | this work |
| 5925 | 630Δ*erm pyrE*^+^*::*P*_tet_-divIVA-(GGGGS)_3_-mScI3 +* pIA33-*ftsZ* | this work |
| 5982 | 630Δ*erm pyrE*^+^*::*P*_tet_-divIVA-(GGGGS)_3_-mScI3 +* pIA33-*sepF* | this work |
| 5926 | 630Δ*erm pyrE*^+^*::*P*_tet_-divIVA-(GGGGS)_3_-mScI3 +* pIA33-*pbp1* | this work |
| 5274 | 630Δ*erm pyrE*^+^*::*P*_tet_-mScI3-(GGGGS)_3_-ftsK* | this work |
| 5928 | 630Δ*erm pyrE*^+^*::*P*_tet_-mScI3-(GGGGS)_3_-ftsK* + pIA33-*ftsZ* | this work |
| 5983 | 630Δ*erm pyrE*^+^*::*P*_tet_-mScI3-(GGGGS)_3_-ftsK* + pIA33-*sepF* | this work |
| 5929 | 630Δ*erm pyrE*^+^*::*P*_tet_-mScI3-(GGGGS)_3_-ftsK* + pIA33-*pbp1* | this work |
| 5270 | 630Δ*erm pyrE*^+^*::*P*_tet_-mScI3-(GGGGS)_3_-pbp3* | (1) |
| 5291 | 630Δ*erm pyrE*^+^*::*P*_tet_-mScI3-(GGGGS)_3_-pbp3 +* pIA33-*ftsZ* | this work |
| 5984 | 630Δ*erm pyrE*^+^*::*P*_tet_-mScI3-(GGGGS)_3_-pbp3 +* pIA33-*sepF* | this work |
| 5293 | 630Δ*erm pyrE*^+^*::*P*_tet_-mScI3-(GGGGS)_3_-pbp3 +* pIA33-*pbp1* | this work |
| 6195 | 630Δ*erm pyrE*^+^*::*P*_tet_*-*mScI3-GSAGSAAGSGKL-*TM *malF_E.coli_* | this work |
| 6193 | 630Δ*erm* Δ*ylmG::aad9* (*ylmG* gene replaced with *aad9* spectinomycin-resistance cassette) | this work |
| 6194 | 630Δ*erm* Δ*ylmH::aad9* (*ylmH* gene replaced with *aad9* spectinomycin-resistance cassette) | this work |
| 5867 | 630Δ*erm* Δ*divIVA pyrE^+^* | this work |
| 5869 | 630Δ*erm* Δ*divIVA pyrE^+^::*P*_cwp2_*-*divIVA* | this work |
| 4502 | 630Δ*erm* Δ*mldA pyrE*^+^ | this work |
| 5863 | 630Δ*erm* Δ*mldA pyrE^+^::* P*_mldA_*-*mldA* | this work |
| 6130 | 630Δ*erm* Δ*mldB pyrE*^+^ | this work |
| 6131 | 630Δ*erm* Δ*mldB pyrE^+^::*P*_mldA_*-*mldB* | this work |
| 6132 | 630Δ*erm* Δ*mldC pyrE*^+^ | this work |
| 6133 | 630Δ*erm* Δ*mldC pyrE^+^::*P*_mldA_*-*mldC* | this work |
| 4024 | 630Δ*erm* Δ*pbp3* *pyrE*^+^ | (2, 4) |
| 5446 | 630Δ*erm* Δ*pyrE* Δ*ftsK::aad9* (*ftsK* gene replaced with *aad9* spectinomycin-resistance cassette) | this work |

**Table S2: *E. coli* strains used in this study.**

| Strain # | Strain or Plasmid Name | Genotype, description, or link to plasmid sequence | source |
| --- | --- | --- | --- |
| 41 | DH5α | F^-^ Φ80*lacZ*ΔM15 Δ(*lacZYA-argF*) U169 *recA1 endA1 hsdR17*(*r_K_*^-^, *m_K_*^+^) *phoA glnV44*(*supE44*) λ^-^ *thi*-*1* *gyrA96* *relA1* |  |
| 531 | HB101/pRK24 | F^-^ *mcrB* *mrr* *hsdS20*(*r_B_*^-^ *m_B_*^-^) *recA13 leuB6 araC14 proA2 lacY1 galK2 xylA5* *mtl*-*1 rpsL20 glnV44*(*supE44*) λ^-^ carrying pRK24 |  |
|  | pIA33-*sepF* | https://benchling.com/s/seq-zgyiqONZtwu7GSmfb1Eg?m=slm-mrRjInfYT0CWlSYX3lhI | this work |
|  | pIA33-*divIVA* | https://benchling.com/s/seq-LOd9trIsngCGXgQOfe1K?m=slm-sSDMTrKL1DJeIaIitlwk | this work |
|  | pIA33-*ftsZ* | https://benchling.com/s/seq-wso4ROJxwgsOYy1EkJMd?m=slm-BdL18Glbu4msmGFBUTfe | this work |
|  | pIA33-*zapA* | https://benchling.com/s/seq-VV3URyIAA8aAURATtDb4?m=slm-V3SsdajBhmK3cXgLIo6W | this work |
|  | pIA33-*pbp1* | https://benchling.com/s/seq-ANaeIGDZzWdeh4ak0Ral?m=slm-H7X6LYBCVxTuisdxnCIc | (2) |
|  | pIA33-neg | https://benchling.com/s/seq-UwWF19ynMbQeQCpa4VZQ?m=slm-omthQY9aQ78LqQgCQRgg | (5) |
|  | pMTL-YN1C | Plasmid to restore *pyrE* and insert cassettes downstream of *pyrE* by allele coupled exchange | (3) |
|  | pMTL-YN1C-P*_tet_*-*ftsZ*-*(GGGGS)_3_-mScI3* | https://benchling.com/s/seq-Z5k9VVOeUVu7tEW60wts?m=slm-E1EyiLGXWLhBNaFgS23X | (1) |
|  | pMTL-YN1C-P*_tet_*-*mScI3*-*(GGGGS)_3_-zapA* | https://benchling.com/s/seq-5wzeu2ioUYnXw2O6RgSP?m=slm-nNMgwsUhMt9hWOtlAWrN | this work |
|  | pMTL-YN1C-P*_tet_*-*sepF*-*(GGGGS)_3_-mScI3* | https://benchling.com/s/seq-BAf3YRk1VkcGS0llMyLz?m=slm-Ms8WaXpKa06nNjihurEW | this work |
|  | pMTL-YN1C-P*_tet_*-*mScI3*-*(GGGGS)_3_-pbp1* | https://benchling.com/s/seq-CJDb9OzLf3RLxFnQwEps?m=slm-GKheauOkSoSxmGbNOKKa | (1) |
|  | pMTL-YN1C-P*_tet_*-*ftsZ*_im_ | https://benchling.com/s/seq-gOxRVYQaoWUTJreTupH9?m=slm-LAJAsoofU7sJ2jZLCRy9 | this work |
|  | pMTL-YN1C-P*_tet_*-*zapA*_im_ | https://benchling.com/s/seq-TKACjeuw9kisIYLXcgN8?m=slm-Cs93ZPj2My7fWbo6zzI0 | this work |
|  | pMTL-YN1C-P*_tet_*-*sepF*_im_ | https://benchling.com/s/seq-CWgldIMYsAKDdX2bY59j?m=slm-aRRZUkmYy0Bq0jzSg6jM | this work |
|  | pMTL-YN1C-P*_tet_*-*pbp1*_im_ | https://benchling.com/s/seq-82isR9GCL5MoMXJH4Swf?m=slm-GuTB57tcE8vY18GGfDOJ | this work |
|  | pMTL-YN1C-P*_tet_*-*ftsZ*_im_-*(GGGGS)_3_-mScI3* | https://benchling.com/s/seq-ABRLEBU2P212BaHlitXR?m=slm-V6h4ayLFlZ1czcRxUywW | this work |
|  | pMTL-YN1C-P*_tet_*-*mScI3*-*(GGGGS)_3_-zapA*_im_ | https://benchling.com/s/seq-rzZjWJAB1IlIjwjz1KbU?m=slm-evZ2gV87c7Di2B8YjwqT | this work |
|  | pMTL-YN1C-P*_tet_*-*sepF*_im_-*(GGGGS)_3_-mScI3* | https://benchling.com/s/seq-iJNpI2zg3kOaTiBomZOU?m=slm-P5MWwcjuShjT34RJgbVa | this work |
|  | pMTL-YN1C-P*_tet_*-*mScI3*-*(GGGGS)_3_-pbp1*_im_ | https://benchling.com/s/seq-KRMemXx4m3TTd0xENFA5?m=slm-TWvGzkl0OUXkcArzYh5C | this work |
|  | pMTL-YN1C-P*_tet_-mScI3-GSAGSAAGSGKL-mldA* | https://benchling.com/s/seq-sPzxSyHj8EoU0Qazfwf0?m=slm-9mQR0FF9CPs5e3Aob0BW | this work |
|  | pMTL-YN1C-P*_tet_-mScI3-GSAGSAAGSGKL-mldB* | https://benchling.com/s/seq-FUMyo2aAmZSllTkX5Mq8?m=slm-TR8q2Vf2ru7nUtTgzBYe | this work |
|  | pMTL-YN1C-P*_tet_-mScI3-GSAGSAAGSGKL-mldC* | https://benchling.com/s/seq-L4jPEHmT8wuG1gPsMsLw?m=slm-woKXjxBHgV9TdgbZ3mIC | this work |
|  | pMTL-YN1C-P*_tet_-divIVA-(GGGGS)_3_-mScI3* | https://benchling.com/s/seq-pET0vDgzaCaXnOaT05pt?m=slm-L0gREnNp31PfQHo5i9Ja | this work |
|  | pMTL-YN1C-P*_tet_-mScI3-(GGGGS)_3_-ftsK* | https://benchling.com/s/seq-u7OtWfwqJpdinc2x0XYl?m=slm-Ux9Hj44AUVWQREgQbqUe | this work |
|  | pMTL-YN1C-P*_tet_-mScI3-(GGGGS)_3_-pbp3* | https://benchling.com/s/seq-AQjfhrx3wGr0vnIsdvlC?m=slm-0Mxy1EJfDMwDuqnZ6Sb2 | (1) |
|  | pMTL-YN1C-P*_tet_*-*mScI3-GSAGSAAGSGKL*-TM *malF_E. coli_* | https://benchling.com/s/seq-bxOUBqqC9vqaO7ju7RJS?m=slm-regg0UDoz1gJyyoScP9C | this work |
|  | pMTL-YN1C-P*_cwp2_*-*divIVA* | https://benchling.com/s/seq-rxTu3fMOhhAmYDcEZuZz?m=slm-cRh0uIfl7ABkQCl1KJm5 | this work |
|  | pMTL-YN1C-P*_mldA_*-*mldA* | https://benchling.com/s/seq-lHgdMTS5LXEgsHYwHI3s?m=slm-dIaGFYQ0GWizxvT298wj | this work |
|  | pMTL-YN1C-P*_mldA_*-*mldB* | https://benchling.com/s/seq-Y53tEy00SIzeoauQyBXB?m=slm-zAjjW93NBLIWqSYaorJZ | this work |
|  | pMTL-YN1C-P*_mldA_*-*mldC* | https://benchling.com/s/seq-KQhrpiCarvlD2vkKwWu1?m=slm-yqcjTDWUazGeOsdUWK0X | this work |
|  | pMTL-YN3 | Plasmid for two-step allelic exchange in Δ*pyrE* *C. difficile* background | (3) |
|  | pMTL-YN3-Δ*mldA* | https://benchling.com/s/seq-JM5ypsHdjKhsyNWkQFZ9?m=slm-HqCFrm14YCHMa3Nk5BQs | this work |
|  | pMTL-YN3-Δ*mldB* | https://benchling.com/s/seq-1YkT9ppPbXnJmMWeBDDK?m=slm-FAg7cNOF0edEZhYNS4cb | this work |
|  | pMTL-YN3-Δ*mldC* | https://benchling.com/s/seq-jsp0Jr8kZAsSdZjCIwF3?m=slm-zpYUw4uNQCcNRk4SmxEN | this work |
|  | pMTL-YN3-Δ*divIVA* | https://benchling.com/s/seq-FISsYsHLHMp1PRRtFwbG?m=slm-xSP23PlY6UGWFe0ARA7w | this work |
|  | pMTL-YN3-Δ*ftsK::aad9* | https://benchling.com/s/seq-CZg13lP3BMmhbEBS7071?m=slm-3py7P8paTpmtsvHxTEqS | this work |
|  | pMTL-YN3-Δ*ylmG::aad9* | https://benchling.com/s/seq-tFVGsG79Oxbd3YLqhQR5?m=slm-G2yOi1JJOyp067EggfGr | this work |
|  | pMTL-YN3-Δ*ylmH::aad9* | https://benchling.com/s/seq-D0A8sbsPc9pK8numroOa?m=slm-Tl104nOWBzPSHxFO6rud | this work |

**REFERENCES:**

1. Harrison GA, Shen A. 2025. Molecular dissection of Class A PBP function uncovers novel features of the non-canonical *Clostridioides difficile* divisome complex. PLoS Genet 21:e1011746-.
2. Shrestha S, Taib N, Gribaldo S, Shen A. 2023. Diversification of division mechanisms in endospore-forming bacteria revealed by analyses of peptidoglycan synthesis in *Clostridioides difficile*. Nat Commun 14:7975.
3. Ng YK, Ehsaan M, Philip S, Collery MM, Janoir C, Collignon A, Cartman ST, Minton NP. 2013. Expanding the repertoire of gene tools for precise manipulation of the *Clostridium difficile* genome: allelic exchange using *pyrE* alleles. PLoS One 8:e56051-.
4. Shrestha S, Harrison GA, Dressler JM, McNellis ME, Shen A. 2025. Penicillin-binding proteins exhibit functional redundancy during asymmetric cell division in *Clostridioides difficile*. J Bacteriol 207:e00503-25.
5. Müh U, Pannullo AG, Weiss DS, Ellermeier CD. 2019. A Xylose-Inducible Expression System and a CRISPR Interference Plasmid for Targeted Knockdown of Gene Expression in *Clostridioides difficile*. J Bacteriol 201:e00711-18.
